# Cartographic privacy: Case studies in communicating sensitive geospatial information

**DOI:** 10.1177/03091325251413610

**Published:** 2026-01-06

**Authors:** David Swanlund, Nadine Schuurman, Michael Martin, Mariana Brussoni

**Affiliations:** 1 Department of Geography, 1763 Simon Fraser University, Burnaby, Canada; 2 School of Environment, 99025 University of Auckland, Auckland, New Zealand; 3 Human Early Learning Partnership, School of Population and Public Health, Faculty of Medicine, The University of British Columbia, Vancouver, Canada; 4 Department of Pediatrics, Faculty of Medicine, The University of British Columbia, Vancouver, Canada

**Keywords:** geoprivacy, privacy, cartography, anonymization, geographic masking, trajectory anonymization, geonarratives

## Abstract

Numerous quantitative techniques exist for protecting geoprivacy, but often require significant expertise and may not always provide adequate protection. We propose cartographic privacy as a framework for protecting sensitive spatial data. Cartographically private approaches favor using design techniques to obfuscate data rather than statistical ones, appealing to intuitive rather than technical understandings of geoprivacy. We explore this concept using two case studies of sensitive geonarrative interviews that focus on mapping the topology and friction of trajectories described by participants, and in doing so protect geoprivacy. We conclude by discussing the advantages and disadvantages of qualitatively driven approaches to geoprivacy protection.

## Introduction & background

The COVID19 pandemic brought Geography to the fore. Contact tracing, a term once reserved for epidemiologists and medical chatter in TV dramas, quickly became a household phrase. As a highly location-based technique, contact tracing aids in containing the spread of disease by asking, *where did a patient go and with whom were they near?* As a result, ordinary citizens have begun to recognize the critical role location data can play in how we manage health crises, along with many other applications in which location data has transformative effects. However, this rise in awareness surrounding location has brought with it increased concern over our locational privacy, often referred to as geoprivacy. As big tech companies bundle software into our devices to aid in background contact tracing, both ordinary citizens and privacy experts alike have voiced their concern about how such spatial data are being recorded, analyzed, and protected (Lehmann, 2020).

Indeed, concerns over geoprivacy are well founded. Location data are often highly sensitive and yet leak continually from our devices. Apps abound that surreptitiously collect our sensitive data and sell them to the highest bidder. For instance, a popular app used by millions of Muslims to calculate prayer times and Qibla was found to be selling users’ locations to the US military (Cox, 2020). There was even internal controversy at Google over how Android phones collect location data despite users reasonably believing that their location services are disabled, with one employee saying about their privacy settings, “The current UI feels like it is designed to make things possible, yet difficult enough that people won’t figure it out” (Lopatto, 2020). In 2023, the FBI even admitted to having purchased location data, ostensibly to circumvent getting a lawful warrant s (Cameron, nd).

It is because of concerns over geoprivacy that many scholars have developed techniques to anonymize location data, of which there are two broad types: single-point locations (e.g., home locations, which are relatively static over time) and space-time trajectories (e.g., GPS movement data). To anonymize single-point locations, GIScientists and epidemiologists have created geographic masks, which subtly alter each location so that it is less likely to reveal sensitive information, such as the home location of cancer patients (Armstrong et al., 1999). In doing so, geographic masks must strike a balance between protecting privacy and ensuring the data remain useful, as too much alteration of the data may result in limited analytical value (Wang et al., 2022). While it is technically possible to use masks to protect individual privacy, the trade-off is that, in some cases, the masking process results in intolerable changes to the data, making them no longer useful or relevant to the analysis. For instance, in a study of highly local environmental factors affecting children’s outdoor play, moving a point even just 50 m might completely alter the context, shifting its location from a park to a school, or a school to an empty lot. In these cases, geographic masks are not always sufficient and limit researchers’ ability to communicate results.

Space-time trajectories feature significantly more descriptive potential than single-point locations, as the increase in spatial context unlocks valuable insights. Transportation planners, for instance, use trajectory data to better assess traffic congestion (Liu et al., 2018; Zheng and Liu, 2017), businesses utilize trajectories to better market towards potential customers (Talbot, 2019), and militaries even use them to identify targets for drone strikes (Franz, 2016; Heller, 2013). However, this added descriptive power also introduces increased potential for privacy violation, and as a result, anonymizing trajectories is far more difficult than single-point locations. Re-identifying a single point location after it has been anonymized is difficult as there are often few reference points that an adversary could use to link that anonymized location to an individual. Trajectories, on the other hand, often feature thousands of such reference points, and yet only four are required to uniquely identify most individuals (Montjoye et al., 2015).

Anonymizing trajectory data therefore requires significant care and expertise. However, further difficulties arise when one begins to consider that the data must still be useful after anonymization; one study found that to fully anonymize a large mobile-phone dataset, locations would have to be altered until they were virtually useless (De Montjoye et al., 2013), a finding that is consistent with similar studies (Krumm, 2007; Montjoye et al., 2015; Zang and Bolot, 2011). As a result, anonymizing trajectory data results in many more cases in which the balance between protecting privacy and ensuring analytical utility cannot be reached (Fiore et al., 2020). A review of trajectory anonymization methods concluded that “mitigation techniques simply do not work” and that “more complex approaches are far from perfect” (Fiore et al., 2020: 140). Moreover, Lin (2024) notes that even well-anonymized datasets can be mined and profiled to the detriment of the groups of people they (indirectly) describe. Together, these pose significant tension between the need for research that is in the public interest and rights to privacy.

In addition to the technical limits of location anonymization, there are also practical barriers to their real-world usage. Kounadi and Leitner (2014) found that among their sample of articles publishing sensitive data in maps, roughly half did not use any geographic masks and published the real sensitive locations. A similar result was found in a study of sexual health journals by Haley et al. (2016). Reasons for this are likely varied, but one barrier is that while geographic masks are often conceptually straightforward, implementing them can be difficult and burdensome, typically requiring coding skills. There has been a recent effort to develop more accessible masking tools, but tools alone are not panaceas (Swanlund, Schuurman and Brussoni, 2020; Swanlund, Schuurman, Zandbergen, et al., 2020). For instance, even with comprehensive tooling, it can be difficult to determine just how much anonymization is enough: 1-in-10, 1-in-100, or 1-in-1000? This is further complicated by the fact that not only is such a figure arbitrary and socially/culturally mediated (Zhang and McKenzie, 2022), but that one can never predict what new datasets will be made public in the future that could be used to link and re-identify individuals (Keßler and McKenzie, 2018). In other words, an anonymized dataset that seems “safe” today could be completely re-identified tomorrow if a seemingly unrelated database leaked. Notably, Differential Privacy is an increasingly popular framework that does offer strong guarantees against such risks, but it is difficult to apply to common geospatial research contexts (Cormode et al., 2012; Dwork and Roth, 2013).

Unfortunately, there is a schism in the literature between anonymization methods meant for single-point data versus trajectories. Methods meant for single-point locations tend to be focused on health and crime research and tend to be authored by those working in GIScience (see for instance Armstrong et al., 1999; Houfaf-Khoufaf et al., 2024; Kounadi and Leitner, 2015; Zandbergen, 2014). Trajectory-based methods are more catered towards real-time data stemming from location-based services, with less attention towards anonymizing data after they have already been collected (as is common in most academic research) (Sila-Nowicka and Thakuriah, 2016). Moreover, these methods lean more towards Computer Science and Engineering (see for instance Chow et al., 2011; Nergiz et al., 2009; Shen et al., 2023; Xue et al., 2013), and, due to the nature of trajectory data, articles tend to be far more complex and less accessible to potential users. This schism may hinder efforts to confront the barriers we have enumerated thus far.

Finally, we are seeing an uptake of location data across a wide range of research. Low-cost sensors are lowering the barrier for studies to begin extensive location tracking in topics ranging from health to marine biology (Almanza et al., 2012; Cullen et al., 2022; He et al., 2023; Yasuda and Arai, 2005). Software platforms are simultaneously making it easier than ever to process and represent location information, evidenced by the rise of the story maps in the digital humanities (Carroll, 2018). This geospatial boom can be both exciting and validating for geographers, but it also poses risks for privacy as the data being collected can be highly revealing. In our own experience, the sensitive nature of location data is often overlooked by those outside the privacy literature, as lat-long coordinates do not immediately stand out as being identifying the way a name or social insurance number does. As such, we hope this article not only encourages geoprivacy researchers to engage more with mixed-methods approaches to privacy protection but also proves instructive for readers that use sensitive geospatial data in their work but have less experience within geoprivacy or GIScience, such as those working in health, crime, or human geography.

Concerned over the coalescence of technical limitations, practical friction, and lack of adoption of quantitative anonymization methods, as well as the uptake of sensitive location data across the academy, this article explores an alternative path towards privacy protection that embraces a qualitative and cartographic approach. This is not to suggest abandoning quantitative solutions, but rather to develop frameworks that guide researchers towards safely visualizing and communicating the *space* and *place* in their data. Indeed, by widening our collective privacy-toolbox to include both quantitative and qualitative techniques, we can better ensure that an appropriate and robust method is available to suit a wider variety of needs. In the next section, we propose a new framework for visualizing sensitive spatial data that relies on alternative representations of space and exemplify these using two case studies of visualizing sensitive trajectory data. Finally, we discuss future research directions for cartographic privacy protection and contextualize its role within the broader geoprivacy literature.

## Conceptualizing cartographic privacy

In 2006, Curtis, Mills, & Leitner discussed the limitations and unknowns surrounding geographic masking, ultimately questioning “are maps really necessary in publications? Why not choose an abstract space on which to display spatial patterns?” These are prudent questions that warrant our attention, and yet they have gone unanswered for nearly two decades. To be clear, much work has been done surrounding abstract data visualization (e.g., Lupi et al., 2016), but not for the specific purpose of privacy protection. Here we revisit Curtis, Mills, & Leitners’ provocation to explicitly unpack alternative, abstract spaces upon which we can *privately* communicate otherwise sensitive geospatial data. We does this using case studies meant to exemplify a different type of “space” in which we can map a given dataset and in doing so achieve *cartographic privacy*.

First, however, we must conceptualize what exactly what we mean by cartographic privacy. A cartographically private map is one that manages to communicate a clear visual message without compromising privacy through the use design choices rather than data manipulation. Cartography has always been a blend of art and science (Robinson and Sale, 1969), but whereas quantitative anonymization leans heavily on its scientific elements, cartographic privacy draws more on artistic decisions. Indeed, rather than relying on quantitative and typically stochastic methods to anonymize a dataset before mapping it, cartographic privacy uses qualitative and typically manual practices to curate changes to the data as it is being mapped. As such, both approaches fundamentally work to transform and obfuscate raw data to produce a map that end-users cannot use to identify individuals, but differ in the methods they use to achieve this. Like in quantitative anonymization, achieving cartographic privacy often means removing variables that are not essential to communicating a clear and concise message while also ensuring that remaining variables cannot be used to identify individuals in the dataset. The latter may be achieved through a variety of means depending on what is to be communicated. For instance, the primary function of transit maps is to communicate the topological relationship between stops, and so the actual locations of those stops are often drastically distorted to favor topological clarity. While privacy is not a concern in this case, we will later illustrate how this topological principle that animates transit maps can be leveraged to map sensitive trajectory data where the nature of and relationships between stops often matters far more than their precise locations.

Producing cartographically private maps is by no means a trivial or straightforward process. Without metrics to measure privacy and information loss, whatever “artistic freedom” that may gained by not being bound to strict quantitative methods is paid for by an increased demand on nuanced qualitative judgment. Sometimes it can be difficult to distill a clear and concise purpose for a map, never mind decide whether it might compromise privacy. This is admittedly a disadvantage of cartographic privacy, but it is important to remember that quantitative methods themselves aren’t immune to subjectivity. Although it is often overlooked, deciding what is a suitable k-anonymity value (a common privacy metric) is a fundamentally qualitative process with no definitive answer (Sweeney, 2002). This problem is magnified when we are often forced to anonymize and release datasets without knowing exactly how they will be analyzed or what data may appear in the future that could enable re-identification.

Nevertheless, the metrics provided by quantitative methods do provide benchmarks to guide analysts’ decision-making, whether they be privacy metrics like k-anonymity or information loss metrics like average displacement distance. For instance, even if an analyst is unsure of whether 30- or 40-anonymity is appropriate, they can still use these metrics to evaluate what anonymization method produces the most privacy for the least information loss. While such metrics are impossible to meaningfully apply to the types of maps produced via cartographic privacy, brief guidelines can be distilled to clarify the design process. Namely, it is worth beginning with the following three questions when designing a cartographically private map:

(1) What is the core spatial relationship that I am trying to convey?

While this question may seem obvious, it can be easy to fall into the trap of designing *general purpose* maps that try to describe the data as completely as possible. Not only does this commonly result in cluttered maps without clear messaging, it is also at odds with cartographic privacy, which requires honing in on one, often *abstract* dimension such that identifying features can be drastically distorted, if not removed entirely. For instance, a project attempting to analyze the inefficiency of ambulance routing may be tempted to publish a general-purpose map of a given ambulance trajectory overlaid with the shortest route. However, this would likely risk patient privacy. Instead, we should focus on the *core spatial relationship*, the degree of deviation between the shortest route and the actual route taken. This may lead us to instead produce a map in which the shortest path is arranged in a straight horizontal line, with the actual route starting at the line’s beginning and arcing around it in a semi-circle towards its end based on the additional travel time; as the additional travel time increases, so does the size of the arc. This would not only protect patient privacy but would arguably provide a more striking representation of just how much time is lost to inefficient routing.

(2) What is the minimally representative map that can adequately convey this message?

One of the simplest and most reliable ways to protect privacy is to reduce the dimensionality of the data. Whether this be through the removal of extraneous variables or the generalization of necessary ones, simplifying the data we wish to represent is a fundamental aspect of cartography that can also greatly reduce its sensitivity (Monmonier, 1991). Therefore, when designing a cartographically private map, it would be prudent to start by exploring what a minimally representative version may look like. In other words, what is the least number of visual variables that are necessary to approximate the relationship we are trying to convey? This is an inexact science and requires a process of exploration (much like in exploratory data analysis) to strike the right balance between being minimal yet representative. Moreover, once that minimally representative version is decided upon, one can often add more detail back in so long as privacy is still preserved. In the previous ambulance routing example, we may decide that we can add more precise distance labels or color the lines based on road type (e.g., highways vs residential streets) without compromising privacy. But by starting from a position of safety and carefully introducing risk, we stand less chance of violating privacy than by starting with a risky map and hoping that our added protections are adequate.

(3) Is there any information in the resulting map that an attacker could use to infer additional information about any individual?

This is the most difficult step in achieving cartographic privacy as it does not require a technical understanding of privacy and vulnerability as much as a conceptual understanding. We differentiate these two as they require drastically different skillsets. In our experience, not everyone who understands how to calculate privacy metrics like k-anonymity and l-diversity fundamentally understands the social and human aspects of *privacy and vulnerability*. In fact, all too often privacy is treated as a mathematical formula to be balanced rather than a deeply social concept and subjective experience (Zhang and McKenzie, 2022). On the other hand, there are those that intimately understand (geo)privacy but lack quantitative skillsets. They may fully understand the *reason* we would use one metric over another but lack the GIS skills to calculate either of them. Cartographic privacy caters to these individuals. Admittedly, this reliance on intuition is simultaneously a strength and weakness of cartographic privacy. And much like most other aspects of research ethics, it is very difficult to define concrete, generalizable rules and methods, as it is idiographic in nature. Nevertheless, there are certainly principles and cautionary signs to be aware of.

The most important of these is uniqueness, which is the antithesis of anonymization. The goal of anonymization is to minimize uniqueness and let the people in our datasets blend into crowds. For instance, by breaking down ambulance routes by incident type, we might find that certain types of incidents only occurred a handful of times. If these include drug overdoses in a small community, people who suspect their neighbors are illicit drug users may be able to use these data to confirm their suspicions. Places are also at the risk of being unique: if an anonymized map of trajectories depicts an unnamed “health clinic,” the consequences of identifying those that visited the clinic would dramatically increase if it was the only clinic in the area and it specialized in substance abuse.

However, there is a large degree of nuance to this, as simply recognizing someone in a dataset is not inherently a privacy violation; someone that recognizes a friend in a dataset because they already know where and when they suffered a drug overdose hasn’t necessarily learned anything new. Worrying about these types of incidents would make anonymization an impossible task. Rather, we should consider privacy to be violated when someone can use that data to *expand their knowledge*.

On the other hand, one can also expand their knowledge using unrelated and often public data. In 2014, the New York Taxi and Limousine Commission released a “de-identified” dataset describing millions of taxi trips, including the time and location of pickup and drop-off. Soon after, Tockar (2014)used the dataset to expose the trip details of celebrities, including where the trip started or ended, and how much they tipped. This was done by finding timestamped photos online of celebrities getting into taxi cabs in New York and then *linking* that to the taxi dataset. Indeed, this is what is known as a *linkage attack*, in which records in an anonymized dataset are re-identified by linking them to other, external datasets (Merener, 2012). Critically, it is difficult, if not impossible, to know what other datasets might exist that could be used for such linkage attacks, particularly given that new datasets may emerge in the future. As such, one should always err on the side of caution when it comes to privacy protection.

Overall, answering these three questions is often an iterative process. A cartographer may find they are able to add additional map elements without compromising privacy, or may find that they need to alter, distort, or entirely remove map elements due to excessive privacy risks. In some cases, a cartographer may even decide that a simple table is the safest method to represent their sensitive data, forgoing a map entirely. We explore this process further in the case studies that follow.

## Case studies of cartographic privacy

This section presents two case studies of cartographic privacy, both involving geonarrative interviews. Geonarratives are spatially linked interviews, often performed while walking a given route, where the participant’s platial descriptions of their environment are mapped (Ajayakumar et al., 2019). This coupling of qualitative and spatial information can form a powerful visualization wherein the descriptive themes are clearly contextualized with locations on a map. Moreover, as Ajayakumar et al. (2019: 1) concisely describe, “these insights can then be used to enrich other more traditional data layers” such that “data gaps can be identified, while more traditional spatial analytical output can be contextualized.” Of course, in interpreting geonarrative maps one must consider that geonarratives are meant to capture the *qualitative experience* of a participant, and as such these maps will inherit all their potential biases about the world around them. The first and most substantial example illustrates how geonarrative interviews of children can be visualized in a transit-style map that emphasizes the *topology* of their chosen route. The second, shorter case study explores how we can visualize the *friction* of a trajectory through hypothetical geonarrative interviews of informal recyclers, referring to often underprivileged people that collect bottles and cans for the deposit.

### Children’s geonarratives

The first example stems from a much larger study that sought to research factors of the natural, built, and social environments that encourage outdoor play in children (Han et al., 2018). The study recruited 105 children aged 10–13 in three separate neighborhoods in Metro Vancouver. Each child wore a GPS tracker for 1 week, drew a map of their neighborhood, and participated in interviews with the researchers. The study was conducted according to the guidelines of the Declaration of Helsinki and approved by the Institutional Review Board of the University of British Columbia.

A significant component of these interviews included a guided walking tour, where children would lead a member of the research team around their neighborhood. These “go-along interviews” were audio-recorded and tracked with a GPS so that they could be transcribed and analyzed (Carpiano, 2009). During the go-along interview children would often point out areas they liked to play in or spend time at, as well as areas they avoided due to boundaries set by their parents or their own safety concerns, among many other topics. To analyze this rich qualitative dataset, we merged the GPS tracks with the transcripts to form geonarratives.

However, geonarratives can also present significant privacy concerns, particularly in this use-case. First, data involving children should *always* be considered with additional care given their vulnerability. This is particularly true in this instance, as children would often describe things that were highly personal, such as the location of bullies. Second, the children’s geographies were extremely localized, making them quite difficult to anonymize from a quantitative perspective. For example, a small group of trees near a child’s house may hold significant meaning to them, making it difficult to perturb or reassign that location while still retaining that meaning. In this regard, they were highly *platial*. Relatedly, the overall range of the children’s mobilities was sometimes incredibly small, such that even small changes to the trajectory would be significant relative to the overall route. Effectively anonymizing these geonarratives would clearly be difficult using quantitative methods alone. For instance, if an abusive parent found out that their estranged child participated in this study, a standard Cartesian map that relied on geographic masking may not provide sufficient protection. This is because while the child’s home could be masked, it is much harder to mask their school, favorite park, or other play spaces without incurring so much information loss that the map becomes no longer useful.

Instead, we aim to explore a more qualitative solution for communicating these sensitive datasets and in doing so illustrate the power of cartographic privacy. More specifically, we will showcase a cartographically private design that highlights the topology of the interview. Our goal is to show how cartographic privacy can protect privacy through more intuitive means and can also sometimes offer clearer insight into certain datasets than standard Euclidean maps. What follows is a description of our thought process in designing these maps, with the intention that readers can learn from, adapt, and expand on it for their own cartographic use cases.

The first step towards mapping these data is to determine what relationship should be conveyed. In this case, the absolute locations of features described in the geonarratives has less importance than the types of features being described, the descriptions themselves, and their location relative to the overall route. For instance, where the participant’s favorite ice-cream shop is within Vancouver is far less important than the fact it is located on their route home from school. Most importantly, this relationship is one of *topology*.

**Figure 1.**
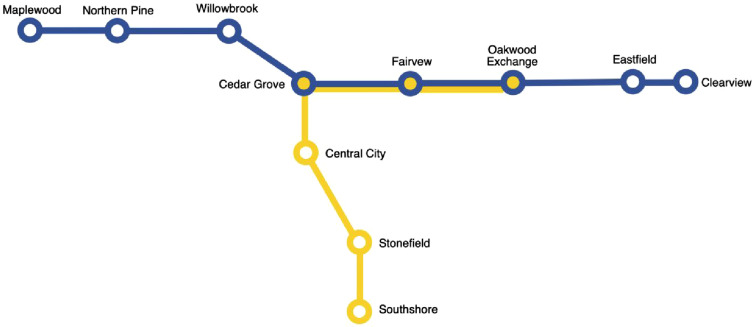
A hypothetical example of a linear cartogram, or transit map, which distorts the actual location of each stop in favor of topological clarity. Each stop represents a node, while the lines between them are edges.

Topology refers to relationships between objects that withstand any amount of stretching or deformation, such as connectivity, containment, and adjacency (Carlson, 2017). Topological relationships like these are fundamental to GIS but also offer significant advantages for creating cartographically private maps. By focusing our map on specific features and their topological relationships, one can begin to not just scale distances or perturb locations, but distort their configuration too, in some cases quite dramatically. This can significantly impede an attacker trying to re-identify participants and their locations. Moreover, topologically focused maps are more familiar than one may imagine. Cartograms, for instance, are a popular style of map that distort map features based on a given attribute while preserving topology, a common example being a world map that grows and shrinks the size of countries based on their relative population. Another type of cartogram, however, is the *linear cartogram*, which are often seen in the form of transit maps (Figure 1). Transit maps heavily distort the geographic position of each stop in favor of clearly depicting routes and connections. Indeed, as Monmonier writes, for transit maps “function dictates form, and a map more ‘accurate’ in the usual sense would not work as well” (Monmonier, 1991: 35). This case study leverages this type of map for privately mapping trajectories.

**Figure 2.**
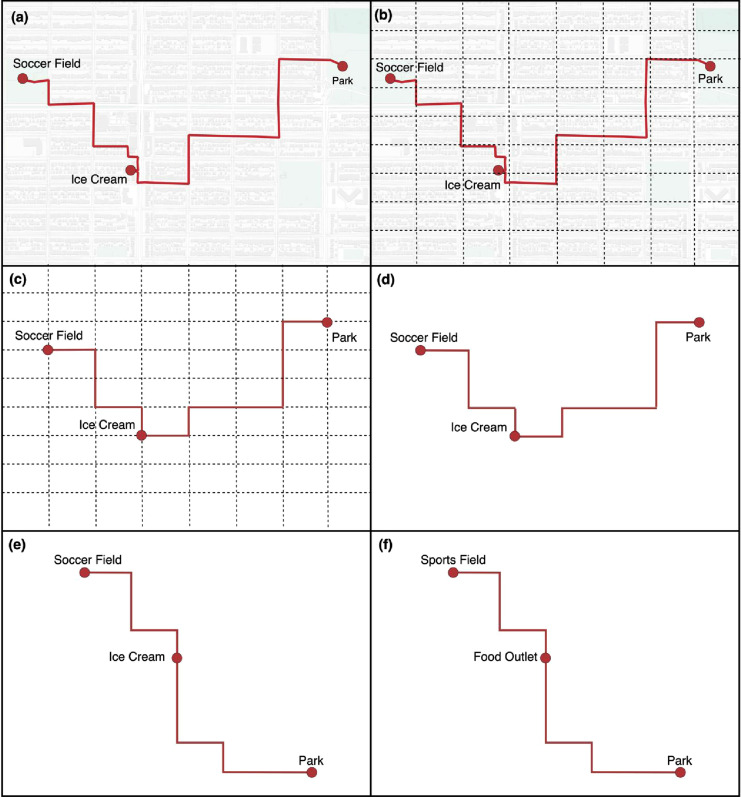
A simple synthetic example showing each step of the design process for cartographic privacy. Panel (a) shows a “original” (fictional) geonarrative, (b) shows the fishnet grid added, roughly sized to the street network, (c) shows the removal of the underlying basemap with points and lines snapped to the grid, and (d) shows the map with the removal of the grid. Finally, (e) shows a reconfiguration of the park further south to further obfuscate the map, while (f) shows the map with generalized feature labels. Basemap is Positron by CartoDB.

In topological terms, the important features along the geonarrative are *nodes*, the participant’s descriptions of them are *attributes*, and the routes connecting them are *edges*. In a purely topological context, it would not matter where the significant features (nodes) are (re)located so long as the routes (edges) between them are preserved. These variables alone would be enough to construct a minimally representative map, as simple straight lines could connect each feature independent of their actual geographical location. However, in this context, we could afford a more relaxed approach, and as such chose to preserve the approximate spatial configuration of each feature (with some exceptions that will be discussed shortly). For instance, if a feature was located a short distance north of another, an approximation of this was preserved in the final map. This was accomplished by overlaying a fishnet grid on top of the geonarrative data before snapping features and routes to the grid. This approach resembles an anonymization technique known as grid-masking (Seidl et al., 2015). Rather than using a specifically defined distance, however, the fishnet grid was sized to roughly align with the gridded street network, creating a coarse grid with a high level of generalization. Routes and features were also manually snapped to the grid using Adobe Illustrator rather than GIS software. Panels (a), (b), and (c) of Figure 2 depicts this process of translating a basic, fictional geonarrative onto the fishnet grid.

Next, we assessed whether the map could expand an attacker’s knowledge base. While the features and the routes connecting them were generalized to a regular grid, it was still possible that the configuration of those features was relatively unique within the city. For instance, Figure 2 shows an ice-cream shop to the south, a soccer field to the north, and a park to the east of the soccer field, this spatial configuration could be unique within the reported study area. Theoretically, a highly motivated attacker could write a script or leverage AI to automate looking for matching configurations in the real world. To mitigate this, we specifically relocated a small number of features such that the park would be further to the south of even the ice cream shop, as shown in panel (e) of Figure 2. In some cases, much larger scale reconfigurations may be necessary. Of course, such modifications incur information loss: while both the topology and approximate distance between each feature along the route have been preserved, the map now depicts the park as being much further from the soccer field. This may or may not matter, depending on whether that proximity is relevant to the overall story and message. If it did, it could be mitigated by removing the fishnet abstraction of the street network and simply showing the route along a straight line, thereby further refining the scope of the message to map readers.

We also slightly adjusted some distances between features, such as by moving a point one cell north on the grid (again, these are only examples, but represent the strategies taken while designing the cartographically private map). While not foolproof (no anonymization is), these small disruptions to the spatial configuration of features helps to mitigate attacks seeking to re-identify data. We should note that this level of protection may not always be needed, but given the sensitivity of our participants and the highly local places they described, our goal was to err on the side of caution and prevent even the neighborhood from being easily identified. Notably, however, this was possible only because we identified our message early on and recognized that knowing the exact geographic locations of the data was in no way essential to understanding the message we were trying to convey.

Finally, attribute data could be added to the map. This included both labels of features participants described as well as select quotes from the interview, both of which require a level of discretion. If a quote contains identifying information, it must of course be anonymized or omitted. Similarly, feature labels must be generalized to not be identifying. This can be as simple as forgoing proper nouns, but in some cases can require more knowledge of the local context. If an area only has one ice-cream shop, then one may need to generalize the label further, such as by calling it “Food Outlet,” as seen in panel (f) of Figure 2. Even then, the remaining parks and sports fields may still be identifying, in which case they may be generalized further to “Green Space.” Indeed, anonymization at this stage required similar sensibilities as any standard privacy protection of qualitative interview data.

**Figure 3.**
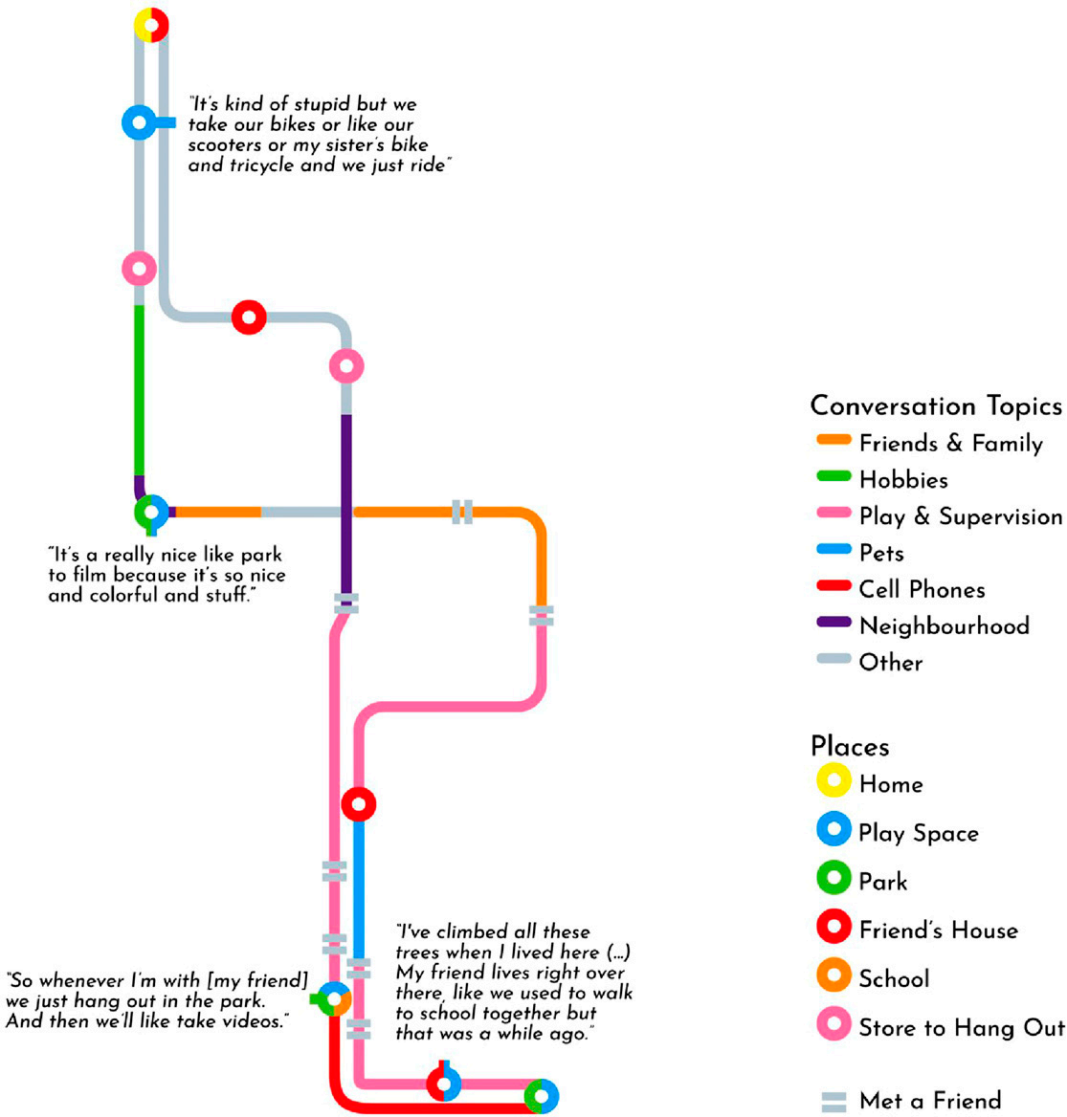
The resulting cartographically private map of a geonarrative go-along interview. The lines of the trajectory are color coded by conversation topic, and notable places along the route are color coded by type. The gridded nature of the lines is due to the underlying street network along which the interview as conducted, which we abstracted such that it was partially preserved.

Figure 3 shows the final transit-style map of a geonarrative interview that includes key features identified by the participant, stylizes lines according to conversation topic, and adds descriptive quotes of each stop where appropriate. We also marked important places that were directly adjacent by utilizing multi-colored symbols, allowing us to easily represent the colocation of, for instance, the participant’s school, a park, and an important play space. One could also utilize this technique to represent contested spaces, such as a favorite play space that becomes unsafe at night. This focus on topology highlights the types of places the participant described, their adjacency, and their connectedness along the participant’s chosen route, while simultaneously describing qualitative themes from the interview. While it does not allow someone to perform GIS analysis, for instance, it takes what otherwise may be plain textual descriptions and breathes life and color into them, offering a perspective on the data that is easy for readers to parse in a privacy-preserving way. Importantly, the map was largely designed using graphic design software and advanced GIS skills were not needed to protect participant privacy.

### Informal recyclers’ routes

While the above transit-style map offers a topological perspective on geonarrative data, there are other characteristics that a cartographically private map may highlight, such as the *friction* of a trajectory. This shorter case study stems from the work of Elliott (2019), who performed walking interviews of informal recyclers: people who collect discarded bottles and cans for recycling in order to collect the deposit. One of Elliott’s findings was that surface texture made a significant impact on some participants, as rougher textures would rattle the shopping carts they use, leading to wrist injuries over time. Elliott, however, was concerned for the participants’ privacy, as many were homeless in a city that was becoming increasingly hostile towards their existence. As such, Swanlund consulted with Elliott on how to best visualize this such that participants’ privacy was sufficiently protected.

**Figure 4.**
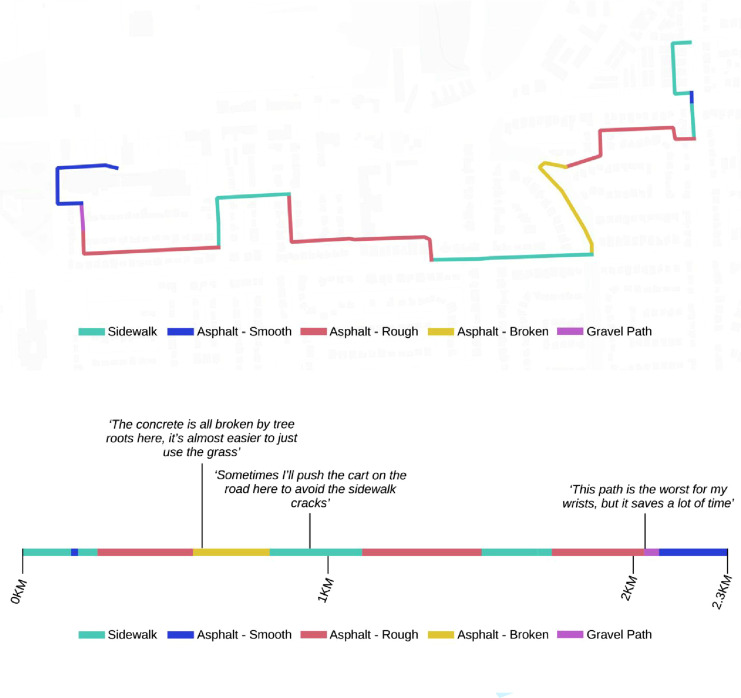
A map of the surface texture of a recycler’s route (top) and the resulting cartographically private visualization (bottom). Note that these data and quotes were synthetically generated for illustrative purposes. In essence, this map becomes cartographically private by removing geographical reference points (e.g., streets and basemaps) and transforming it from having two spatial dimensions (*x,y*) to only one (*x*). Basemap is Positron by CartoDB.

Despite not having training in data privacy, Elliott leveraged her *intuitive* understanding of privacy and discussed essentially using linear referencing to create a straight-line visualization of the road and sidewalk surface texture along the route, similar to a timeline graphic but based on distance. While the graphic was ultimately not produced, we saw this as an excellent example of cartographic privacy and have attempted to illustrate it here by synthesizing a geonarrative of a fictional recycler (Figure 4). As these data are entirely synthetic, we are able to include a “before” visualization as well in the top half of the graphic. To achieve this, we created a trajectory that was broken up into segments according to surface texture. We then calculated the distance of each segment and divided it by the total trajectory distance, giving the percent distance of each surface texture. We then used graphic design software to create a line 100 units long, and overlaid each segment on top of it (e.g., a sidewalk segment that was 4% of the trajectory’s distance was represented as a line 4 units long). Finally, we added distance markers and a legend, and wrote illustrative quotes to complete the geonarrative example.

This cartographically private visualization provides viewers with a sense of the friction along the recycler’s routes without directly exposing the route itself. To provide additional protection, distances could be generalized slightly to protect against re-identification attacks using road segment lengths. Nevertheless, this type of visualization represents a relatively simple yet effective solution to what would otherwise be a difficult anonymization problem using quantitative methods alone.

## Discussion & conclusion

When given the task of communicating sensitive data, it is unsurprising that we often default to quantitative thinking. Methods abound that employ sophisticated techniques to obfuscate and synthesize datasets in the name of privacy protection (Clarke, 2016; Houfaf-Khoufaf et al., 2024; Lin, 2023; Seidl et al., 2016; Zurbarán et al., 2018). Quantitative privacy methods are ideal when the goal is to share a dataset that other data-scientists can analyze, and they indeed have many advantages; the ability to quantify both privacy protection and information loss is undeniably valuable. However, these need not, and should not, be the *only* tools in our privacy toolbox. Geospatial data can be a powerful tool in qualitative and mixed-methods research where the goal is not to share an analyzable dataset, but rather to communicate *meaning* (Cope and Elwood, 2009). It is here that cartographic privacy makes its home.

A core principle of much geographic research is that space must be considered from more than just Euclidean perspectives. In fact, this very concept fueled the critical GIS debates of the 1990s and ultimately led to the development of qualitative GIS (Cope and Elwood, 2009; Schuurman, 2000). And yet, as Bergmann and Lally (2021) illustrate, very little has actually been done to integrate these alternative spaces into GIScience, never mind GISystems. We see this article as one small step towards this larger project of expanding the practice of GIS beyond the mostly Euclidean spaces to which it has thus far been confined.

An added benefit of cartographically private approaches is that they are less likely to result in false attribution. This occurs when a map reader does not understand that locations in the map were anonymized, leading them to incorrectly attribute information about an anonymized point (Seidl et al., 2018). For instance, if a point describing the home location of a cancer patient is randomly perturbed and displaced to another address, false attribution occurs when the map reader assumes that the person at the anonymized address has cancer. Seidl et al. (2018) propose Voronoi masking as a solution to this, but cartographically private maps have the potential to provide much stronger protection from false attribution. Indeed, in our geonarrative visualization, false attribution is virtually impossible.

Moreover, a key concept in quantitative anonymization is information loss, referring to loss of a dataset’s analytical value through the process of being anonymized. This is, of course, also an issue with cartographic privacy, though is more difficult to grapple with given the more qualitative, idiographic nature of the approach. Certainly, by excluding certain geospatial context, such as road networks and nearby points of interest, the efficacy of some geonarrative maps may be compromised. Nevertheless, future research into cartographic privacy could identify common design patterns, such as the transit map for representing geonarratives, and conduct usability testing to assess whether they remain effective communication tools. In other words, what conclusions would a map-reader come to if shown the original, unprotected geonarrative map as compared to the heavily obfuscated version? This research could offer improved design guidelines for creating cartographically private maps, similar to Leitner & Curtis’ empirical research into geographic masking techniques and their own resulting cartographic guidelines (Leitner and Curtis, 2004).

A potential downside to cartographic privacy is that people who attempt to create cartographically private maps may be more likely to cause privacy violations due to a lack of tools to quantify risk or expertise in privacy protection. While this is a valid criticism, it must be noted that this is likely already happening. Even with metrics like k-anonymity and displacement distance, it is difficult to interpret what is an acceptable value, as it is not only dependent on context, but is also largely socially mediated. Moreover, while geoprivacy scholars have devised many methods for privacy protection, they have been far more hesitant to provide tangible guidelines as to what level of privacy protection is acceptable. One could read the entire geographic masking literature and still not know whether to aim for k-anonymity values of 10 or 100. To make matters worse, studies have found that many maps entirely lack anonymization of sensitive information (Haley et al., 2016; Kounadi and Leitner, 2014). Therefore, while we do acknowledge the ambiguities within cartographic privacy, our goal in writing this paper is to at the very least provide a level of *harm reduction*. Nevertheless, we recommend that readers applying these techniques always err on the side of caution, and if the data is extremely sensitive or high risk, consider forgoing a map altogether.

Another valid criticism of cartographic privacy is that it blurs the lines between cartography and data visualization more generally. One may ask at what level of spatial abstraction does a map stop being a map? This is once again a fair question, but also somewhat misses the point. Maps will always have varying degrees of spatiality; the first transit-style “tube map” created by Harry Beck was “initially rejected by […] because it was considered too radical,” but today these topologically oriented maps are commonplace and hardly controversial (Transport for London, nd). While cartographically private designs may sometimes veer more into data visualization than cartography, we would ask, *so what?* The point of cartographic privacy is not to demarcate what is and is not a map, but rather to provide a framework that helps guide cartographers to a suitable method of visually communicating sensitive spatial information. Sometimes their results may indeed end up looking more like standard data visualizations than maps, and *that is fine*. Our concern is over the *substance* of geoprivacy protection rather than the label that describes how we achieve that protection.

We also want to make clear we in no way suggest that cartographers have not wrestled with geoprivacy before; surely many cartographers have thought critically about what sensitive information their maps could expose and have worked diligently and creatively to protect geoprivacy. However, this has largely remained hidden in the everyday workings of map production, and as a result there has been little formalization of both problems and solutions in the literature. This article attempts to perform that necessary step of formalization and prompt a more explicit, focused discussion that brings geoprivacy protection via cartographic design to the fore (Schuurman, 2006).

Ultimately, for readers within the geoprivacy space, we hope that by exploring these case studies we have sparked a curiosity for how else we may *(1)* communicate sensitive geospatial insights, *(2)* address geoprivacy concerns within mixed-methods research, and *(3)* consider the *users* of geoprivacy techniques. In working on geoprivacy protection, we must consider the real-world, practical application of our work; while it is necessary and worthwhile to develop robust quantitative, data-oriented solutions, we must also consider other use cases where geoprivacy deserves consideration. For readers outside of the geoprivacy literature, we hope this article has *(1)* conveyed the need for geoprivacy considerations beyond just data-oriented fields like GIScience, *(2)* demonstrated that communicating sensitive geospatial information need not be a highly technical endeavor, and *(3)* provided enough guidance to begin designing cartographically private visualizations that communicate the *meaning* in your work rather than the coordinates.

## Data Availability

Data cannot be shared due to privacy concerns.*
